# Qing-Xin-Jie-Yu granule prevents myocardial infarction-induced apoptosis via inhibition of p38 MAPK pathway

**DOI:** 10.3389/fcvm.2026.1617408

**Published:** 2026-01-30

**Authors:** Jianghan Qi, Qiaoyan Cai, Ying Han, Xiaoyao Gao, Meiling Yang, Chenyi Wei, Keyi Wang, Zhenghao Lyu, Yuxing Lin, Ling Zhang, Zhuye Gao, Jianfeng Chu

**Affiliations:** 1 College of Integrative Medicine, Academy of Integrative Medicine, Fujian University of Traditional Chinese Medicine, Fuzhou, Fujian, China; 2 Fujian Key Laboratory of Integrative Medicine on Geriatrics, Fujian University of Traditional Chinese Medicine, Fuzhou, Fujian, China; 3 Chen Keji Academic Thought Inheritance Studio, Fujian University of Traditional Chinese Medicine, Fuzhou, Fujian, China; 4 Department of Acupuncture and Moxibustion, The Third Affiliated Hospital of Fujian University of Traditional Chinese Medicine, Fuzhou, Fujian, China; 5 Cardiovascular Diseases Center, Xiyuan Hospital, China Academy of Chinese Medical Sciences, Beijing, China

**Keywords:** apoptosis, myocardial infarction, network pharmacology, p38 MAPK pathway, qing-Xin-Jie-Yu granule

## Abstract

**Background and aim:**

Myocardial infarction (MI) is a leading cause of death globally, and traditional Chinese medicine (TCM) offers therapeutic potential through its multi-targeted approach. This study aims to investigate the protective mechanisms of Qing-Xin-Jie-Yu Granule (QXJYG) against MI.

**Methods:**

Network pharmacology was carried out to predict targets and pathways of QXJYG in the treatment of MI. An *in vivo* mouse model of MI was induced via left anterior descending coronary artery ligation, and hypoxia-induced H9C2 cells were performed as the *in vitro* model. Cardiac function was assessed by echocardiography, while histological changes were analyzed using HE and Masson's trichrome staining. The positive expression of CD31 was used to assess microvascular density in the hearts of MI mice via immunohistochemistry. Serum levels of superoxide dismutase (SOD), lactate dehydrogenase (LDH), and malondialdehyde (MDA) were evaluated, alongside LDH and MDA in cell culture supernatants. Apoptosis in cardiac tissue was detected by TUNEL staining, while apoptosis in hypoxia-induced H9C2 cells was assessed using Annexin V/PI and Hoechst 33,258 staining. Western blot analysis was conducted to evaluate the protein expression of p-p38 MAPK, p38 MAPK, Bcl-2, Bax *in vivo* and *in vitro* experiments.

**Results:**

GO and KEGG analyses suggest that QXJYG affects potential targets related to cellular oxidative stress and apoptosis in MI. *In vivo*, QXJYG effectively enhanced cardiac function, increased microvessel density, lowered serum LDH and MDA levels, elevated serum SOD levels, and reduced apoptosis in cardiac tissue. It also suppressed the ratio of p-p38 MAPK/p38 MAPK, downregulated Bax protein expression, and upregulated Bcl-2 protein expression in MI mice. *In vitro* experiments revealed that QXJYG decreased LDH and MDA levels in the culture supernatants, reduced hypoxia-induced apoptosis, inhibited the ratio of p-p38 MAPK/p38 MAPK, decreased Bax protein expression, and enhanced Bcl-2 protein expression. The p38 MAPK inhibitor SB203580 and QXJYG combination more effectively reduced MDA levels and apoptosis in hypoxia-induced H9C2 cells than QXJYG alone.

**Conclusions:**

This study demonstrates that QXJYG exerts cardioprotective effects against MI by improving cardiac function, reducing oxidative stress, and inhibiting apoptosis through the regulation of the p38 MAPK signaling pathway both *in vivo* and *in vitro*.

## Introduction

Myocardial infarction (MI) is a critical cardiovascular event caused by the heart's insufficient regenerative capacity, resulting in irreversible loss of cardiomyocytes. The frontiers of regenerative medicine, including stem cell and engineered extracellular vesicle therapies, have opened new pathways for the recovery from MI (1, 2). Yet, issues such as the myocardial no-reflow phenomenon and reperfusion injury persist, contributing to the high mortality rates observed during acute MI hospitalization. The quest for more effective therapies remains an urgent priority in the field of cardiology.

Traditional Chinese medicine (TCM) has a long history in the treatment of coronary heart disease (CHD) due to its multi-component and multi-target characteristics. Under the leadership of Academician Keji Chen, researchers have integrated traditional TCM principles with modern medical advances to propose the innovative “blood stasis and toxin causing catastrophe hypothesis” (3). Based on this theory, they developed Qing-Xin-Jie-Yu Granule (QXJYG), a compound formulation composed of *Astragalus membranaceus*, *Salvia miltiorrhiza Bunge*, *Ligusticum striatum*, *Agastache rugosa*, and *Coptis chinensis*. QXJYG is widely used clinically for CHD treatment, regulating inflammatory cytokine levels, improving hemorheological properties, and cardiac function indices in patients. It slows the progression of stable coronary artery disease, thereby reducing cardiovascular events (4–7). However, its specific therapeutic mechanisms remain incompletely elucidated.

Network pharmacology, an emerging field integrating systems biology and bioinformatics, can comprehensively reveal the molecular, cellular, tissue, and organ-level mechanisms of TCM formulations. Therefore, we used network pharmacology to analyze drug targets and key pathways of QXJYG in the treatment of MI. Additionally, both *in vitro* and *in vivo* experiments were performed to confirm the predictions synthesized by network pharmacology.

## Materials and methods

### Collection of active ingredients and potential targets of QXJYG

Utilize the Traditional Chinese Medicine Systems Pharmacology Database and Analysis Platform (TCMSP, https://old.tcmsp-e.com/index.php) to search for the chemical components of *Astragalus membranaceus* (Huangqi), *Salvia miltiorrhiza Bunge* (Danshen), *Ligusticum striatum* (Chuanxiong), *Agastache rugosa* (Huoxiang), and *Coptis chinensis* (Huanglian) in QXJYG. Based on pharmacokinetic properties such as absorption, distribution, metabolism, and excretion, screen for active ingredients using the criteria of oral bioavailability (OB) >30% and drug likeness (DL) >0.18. Obtain the isomeric SMILES of QXJYG-related active ingredients from the PubChem website (https://pubchem.ncbi.nlm.nih.gov/). Use the obtained SMILES to search on the SwissTarget Prediction website (http://www.swisstargetprediction.ch) to predict the potential targets of the active ingredients. All predicted targets with a non-zero probability were retained and considered as potential targets of the compounds.

### Prediction of potential targets for QXJYG in the treatment of MI

Retrieve and filter potential targets related to MI through the DisGeNET database (https://www.disgenet.com) and the GeneCards database (https://www.genecards.org). Remove duplicate targets after merging the databases to obtain the related targets of MI. Use Venny 2.1.0 (http://www.liuxiaoyuyuan.cn) to identify the intersection targets between MI and the potential targets of QXJYG, and construct a Venn diagram. Import the screened active ingredients of QXJYG and the intersection targets from the Venn diagram into Cytoscape 3.10.0 to construct a “compound-target-disease” network map.

### Protein-protein interaction (PPI) and gene enrichment analysis

Import the intersecting genes of QXJYG and MI into the STRING network platform (https://string-db.org) and select the species “Homo sapiens”. Set the confidence score to >0.4 as a condition for screening and generate the interaction relationships of the intersection targets. Import this network diagram into Cytoscape 3.10.0 for analysis. Use the CentiScape 2.2 plugin to calculate the degree and betweenness of each node. Identify the key nodes as those with degree and betweenness values higher than the average of these two metrics. The intersection targets were subjected to gene ontology (GO) and Kyoto Encyclopedia of Genes and Genomes (KEGG) pathway enrichment analyses using the DAVID platform (https://david.ncifcrf.gov/summary.jsp). GO functional enrichment was categorized into three domains: biological processes (BP), cellular components (CC), and molecular functions (MF). The KEGG pathway enrichment analysis aimed to elucidate the underlying mechanisms by which QXJYG exerts its effects on MI. The resulting data were visualized using bar charts and bubble diagrams, created with the assistance of the micro-information visual cloud platform (https://www.bioinformatics.com.cn).

### Preparation of experimental drug

QXJYG was prepared and provided by Jiangyin Tianjiang Pharmaceutical Co., Ltd (Jiangsu, China; batch number: 2112308). The product is a water-soluble granular formulation manufactured through standardized extraction, purification, and spray-drying procedures. The manufacturer conducted a multi-indicator quality control assessment and established UPLC fingerprints for QXJYG. Details regarding its preparation, chemical profiling, and quantification of active constituents have been previously published (8–10). We used the same batch of QXJYG as in the previous studies (9). Isosorbide mononitrate (ISMN; MedChemExpress, USA) was prepared in 0.9% saline for animal use and in sterile PBS for *in vitro* experiments. Stock solutions were stored at −80 ℃ and diluted with serum-free DMEM prior to use. SB203580 (HY-10256, p38 MAPK inhibitor; MedChemExpress, USA) was dissolved in DMSO to prepare a 10 mM stock solution and diluted to 10 μM for cell treatment. This concentration was selected based on previous studies that demonstrated effective inhibition of p38 MAPK activity (11).

### Animals and grouping

Male C57BL/6 mice (10–12 weeks old, 20–22 g) were purchased from Shanghai Slack Experimental Animals Co., Ltd. All animals were housed under standard laboratory conditions at 24 ± 2  °C with 50%–60% relative humidity and a 12-h light/dark cycle. Mice were acclimatized for 5 days prior to experimentation. All animal procedures were reviewed and approved by the Animal Ethics Committee of the Research Institute at Fujian University of Traditional Chinese Medicine (FJTCM IACUC 2022197) and conducted in accordance with institutional guidelines and internationally accepted principles for the care and use of laboratory animals.

A total of 48 mice were randomly assigned into six groups (*n* = 8 per group) based on body weight**.** The dosages of QXJYG and ISMN were calculated from their respective adult clinical doses and converted to mouse-equivalent doses using a body surface area -based method, with the converted QXJYG dose defined as the medium dose and the low and high doses set at half and twofold of the medium dose, respectively. The mice were divided into the following groups: ① Sham (received saline, ig), ② Model (MI, received saline, ig), ③ ISMN (MI+5.2 mg/kg/day ISMN, ig), ④ QXJYG-L (MI+1.43 mg/g/day QXJYG, ig), ⑤ QXJYG-M (MI+2.86 mg/g/day QXJYG, ig), and ⑥ QXJYG-H (MI+5.72 mg/g/day QXJYG-H, ig). Drug administration was initiated on the first day after MI surgery and continued once daily for four weeks. The Sham and Model groups received normal saline, while the other groups received either ISMN or QXJYG at the indicated doses.

### Mouse model of MI

Mice subjected to left anterior descending branch (LAD) coronary artery ligation was performed to establish the model of MI following the methodology previously described by Shen et al. (12). Briefly, mice with pre-plucked chest hair were anaesthetised with 1% isoflurane gas at a flow rate of 0.6–1 L/min, and ligated 2–3 mm below the left auricle with a sterile non-absorbable banded suture needle, size 6–0. Surgical procedures for the Sham group mirrored those of the Model group, with the exception that no ligation was executed.

### Cardiac echocardiography

Cardiac function in mice was assessed after a 4-week treatment period using the high-resolution Vevo2100 ultrasound system. Following chest hair removal, the mice were anesthetized with isoflurane and positioned supine on an animal operating platform. The MS400 ultrasound probe was oriented at a 45 ° angle relative to the midline on the left side of the sternum to capture a long-axis view of the left ventricle. Parameters of cardiac function, such as ejection fraction (EF) and fractional shortening (FS), were evaluated using Vevo LAB offline software.

### Sample collection

After four weeks of drug intervention, mice were euthanized following blood collection. The hearts were then removed, photographed, and cut longitudinally into two halves, with one half containing the entire apex region and the other half containing the entire base region. The apical portion was washed with saline, fixed into a 4% paraformaldehyde solution for 48 h, and then embedded with paraffin for histological analysis. The remaining half of the heart specimens designated for western blot analysis were frozen at −80 ℃. The blood was centrifuged (4 ℃, 3,000 rpm, 15 min) to separate the serum, which was then stored at −80 ℃ for subsequent experiments.

### Histopathological analysis

Paraffin-embedded sections (4 μm thick) were stained with hematoxylin and eosin (H&E) and Masson's trichrome (Solarbio, Beijing, China) to visualize myocardial pathology and assess collagen volume fraction (CVF) for fibrosis quantification, respectively. Examination and imaging were performed using an optical microscope (Leica, Germany) at 400× magnification, with CVF analysis conducted via ImageJ software (NIH, USA).

For immunohistochemical staining, antigen retrieval was performed on mouse heart sections using 0.01M sodium citrate buffer (MVS-0066, Maixin, Fuzhou, China). Sections were incubated with CD31 antibody (1:200 dilution, 28083-1-AP, Proteintech Group, Inc, China) overnight at 4 ℃, followed by washing with PBS and secondary antibody incubation with HRP-polymer-conjugated anti-Mouse/Rabbit IgG (Maixin, KIT-9921, Fuzhou, China). Visualization was achieved using a DAB kit (Maixin, DAB-2031) and hematoxylin counterstaining. Slides were observed under a light microscope at 400× magnification, and positive expression was analyzed using ImageJ software in six random fields of view.

### TUNEL assay

Apoptosis in myocardial tissue was detected using a TUNEL assay kit (MK1025, Boster, Wuhan, China) following the manufacturer's protocol. Briefly, sections were treated with 3% hydrogen peroxide to inactivate endogenous peroxidases, followed by Proteinase K digestion. DNA fragmentation was labeled using terminal deoxynucleotidyl transferase (TdT) and DIG-dUTP, with signal amplification achieved through biotinylated anti-DIG antibodies and a streptavidin-HRP complex. Visualization was performed with DAB and hematoxylin counterstaining. Positive cells were quantified in randomly selected fields using ImageJ software.

### Enzyme-linked immunosorbent assay

The collected serum samples were utilized for enzyme-linked immunosorbent assay (ELISA) to measure the levels of various cardiac biomarkers. Specifically, the ELISA kits were conducted to detect the levels of Superoxide Dismutase (SOD, catalog number U96-3518E), Lactate Dehydrogenase (LDH, catalog number MM-44145M1), respectively. These ELISA kits were procured from U-BioA (Shanghai) Trading Co., Ltd. China. The ELISA detection procedure was performed in strict accordance with the guidelines provided in the respective ELISA kit instructions.

### Determination of MDA and LDH levels

To assess oxidative stress and cell injury, MDA levels were measured in both serum and cell culture supernatants, while LDH release was analyzed in cell supernatants. MDA levels were determined using a thiobarbituric acid (TBA) reaction-based assay (ZC-S0343, Shanghai Zcibio Technology Co., Ltd., China). Briefly, samples were incubated with TBA reagent in a boiling water bath, allowing MDA to react with TBA and form a red-colored complex. After cooling, the absorbance was measured at both 532 nm and 600 nm using a spectrophotometer. The difference in absorbance at these two wavelengths was used to calculate MDA levels, following the manufacturer's instructions.

LDH activity was quantified using a commercial LDH assay kit (A020-2-2, Nanjing Jiancheng Bioengineering Institute, China). Following the manufacturer's instructions, samples were incubated with reaction reagents at room temperature. Absorbance was measured at 450 nm using a microplate reader, and LDH activity was calculated according to the standard curve provided with the kit.

### *In vitro* culture of the H9C2 myocardial cell line

H9C2 cells (Rattus norvegicus, CVCL 0286, SCSP-5211) were cultured in DMEM with 10% FBS and 1% Penicillin-Streptomycin (100×) at 37 ℃, 5% CO_2_, and saturated humidity until 80%–90% confluence for passaging and seeding. For experiments, cells were seeded at 10^3^ cells/well in 96-well plates and treated with a range of concentrations of QXJYG or ISMN for the CCK-8 assay to determine non-cytotoxic and effective concentrations. Based on the results of cell viability, the selected concentrations of QXJYG (low, medium, and high) and ISMN were used for subsequent *in vitro* experiments. Then, they were seeded at 8 × 10^4^ cells/well in 6-well plates for western blot or qPCR experiments, grouped as follows: ① Control (normoxia); ② Model (hypoxia); ③ ISMN (hypoxia+900 μM ISMN); ④ QXJYG-L (hypoxia+7.5 μg/mL QXJYG); ⑤ QXJYG-M (hypoxia+15 μg/mL QXJYG); ⑥ QXJYG-H (hypoxia+30 μg/mL QXJYG).

Meanwhile, cells were divided into five groups: ① Control (normoxia); ② Model (hypoxia); ③ QXJYG (hypoxia + 30 μg/mL QXJYG); ④ SB203580 (hypoxia + 10 μM SB203580); ⑤ QXJYG + SB203580 (hypoxia + 30 μg/mL QXJYG + 10 μM SB203580). Oxidative stress and apoptosis levels in each group were assessed using MDA assay kits and Hoechst 33,258 staining. After 24 h of plating, cells were treated with medication and subjected to 24 h of hypoxia according to their respective groups, the anoxic conditions were based on previous laboratory data: 1%O_2_, 94% N_2_ and 5% CO_2_. For the SB203580 group, cells were pretreated with 10 μM SB203580 for 1 h, after which the medium was replaced with fresh DMEM before hypoxia. For the QXJYG + SB203580 group, cells were pretreated with 10 μM SB203580 for 1 h, followed by medium replacement with DMEM containing 30 μg/mL QXJYG prior to hypoxia. Following the completion of hypoxic culture, cells and the corresponding culture medium are harvested for further experimental procedures.

### Cell counting Kit-8 assay

Cell viability was evaluated using the Cell Counting Kit-8 (CCK-8; Abbkine, USA; Cat. KTC011001). The H9C2 cells were seeded into 96-well plates and allowed to adhere for 24 h, followed by treatment with various concentrations of QXJYG or ISMN for an additional 24 h. After incubation, 10 μL of CCK-8 reagent was added to each well, and cells were further incubated for 1 h at 37 °C. The optical density (OD) was measured at 450 nm using a microplate reader. Cell viability was calculated based on OD values, and the results were used to determine the optimal concentrations of QXJYG and ISMN for subsequent H9C2 cell intervention experiments.

### Western blot analysis

Western blot analysis was performed to detect protein expression in heart tissue and H9C2 cells. Samples were homogenized and lysed in Western & IP Lysis Buffer (P0013, Beyotime, China) supplemented with protease inhibitor cocktail (HY-K0010, MedChemExpress, USA), phosphatase inhibitor (PhosSTOP, 04906845001, Roche Diagnostics GmbH, Germany), and phenylmethylsulfonyl fluoride (PMSF, P0100-10, Beijing Solaibao Technology Co., Ltd., China). Lysates were incubated on ice for 15 min and centrifuged at 14,000× g for 15 min at 4 °C. Protein concentrations were determined using a BCA protein assay kit (23225, Thermo Fisher Scientific, USA).

Equal amounts of total protein (40 μg) were separated by 10% SDS-PAGE and transferred to 0.45 µm PVDF membrane (AmershamHybond, GE Healthcare, Munich, Germany). Membranes were blocked with a rapid protein-free blocking solution (PS108P, Shanghai Yemei Bio-Medical Technology Co., Ltd.) for 30 min at room temperature, and then incubated overnight at 4 ℃ with the following primary antibodies [rabbit anti-p38 MAPK (1:1,000 dilution, #8690, Cell Signaling Technology, USA), rabbit anti-Phospho-p38 MAPK antibody (1:1,000 dilution, #9,211, Cell Signaling Technology, USA), rabbit anti-Bcl-2 (1:1,000 dilution, #3498, Cell Signaling Technology, USA), rabbit anti-Bax (1:1,000 dilution, #2772, Cell Signaling Technology, USA), mouse anti-β-Actin (1:10,000 dilution, 81115-1-RR, Proteintech Group, Inc, China) and rabbit anti-Vinculin (1:5,000 dilution, GTX113294, GeneTex, USA)]. After incubation, the membrane was washed with TBST and then incubated with secondary antibodies [rabbit secondary antibody (L3012, SAB, USA) and mouse secondary antibody (L35665, SAB, USA)] at room temperature for 1 h. Following incubation, the membrane was washed with TBST and then detected with an ECL detection kit (Thermo Fisher Scientific, USA). The band intensities were analyzed using ImageJ software.

### Flow cytometry assay

To assess the rate of cell apoptosis, H9C2 cells in each group were harvested and stained using the Annexin V/PI staining kit (KGA107, Jiangsu Kaiji Biotechnology Co., Ltd., Nanjing, China) following the manufacturer's instructions, and analyzed by flow cytometry (BD Biosciences, Franklin Lakes, NJ, USA).

### Assessment of apoptosis via hoechst 33258 staining

Apoptosis in H9C2 cells was assessed by examining nuclear morphological changes using the Hoechst Staining Kit (C003, Shanghai Beyotime Biotechnology Co., Ltd., China). Normal cell nuclei appeared blue, while apoptotic cell nuclei displayed a dense bright color, either densely stained or fragmented. Using ImageJ software to count the numbers of apoptotic (bright) and non-apoptotic (blue) nuclei. The apoptosis index was calculated as follows: Apoptosis rate = (apoptotic nuclei count/total nuclei count) × 100%.

### Statistical analysis

All data were analyzed using SPSS 23.0. For metric data that conform to a normal distribution, the mean ± *SD* was used for representation. For non-normally distributed data, we reported the median and interquartile range. Normality of the metric data was assessed using the Shapiro–Wilk test. If data were normally distributed, we used one-way ANOVA to compare multiple groups, followed by *post-hoc* analysis with the LSD test for homogeneous variances and the Games-Howell test for heterogeneous variances. Nonparametric analysis (Kruskal–Wallis One-Way ANOVA) was conducted for data that did not conform to a normal distribution. The Bonferroni method was used to adjust for significance in cases of multiple comparisons. And the results were considered statistically significant when *P* < 0.05.

## Results

### Potential therapeutic mechanisms and compounds of QXJYG against MI

**Figure 1:**
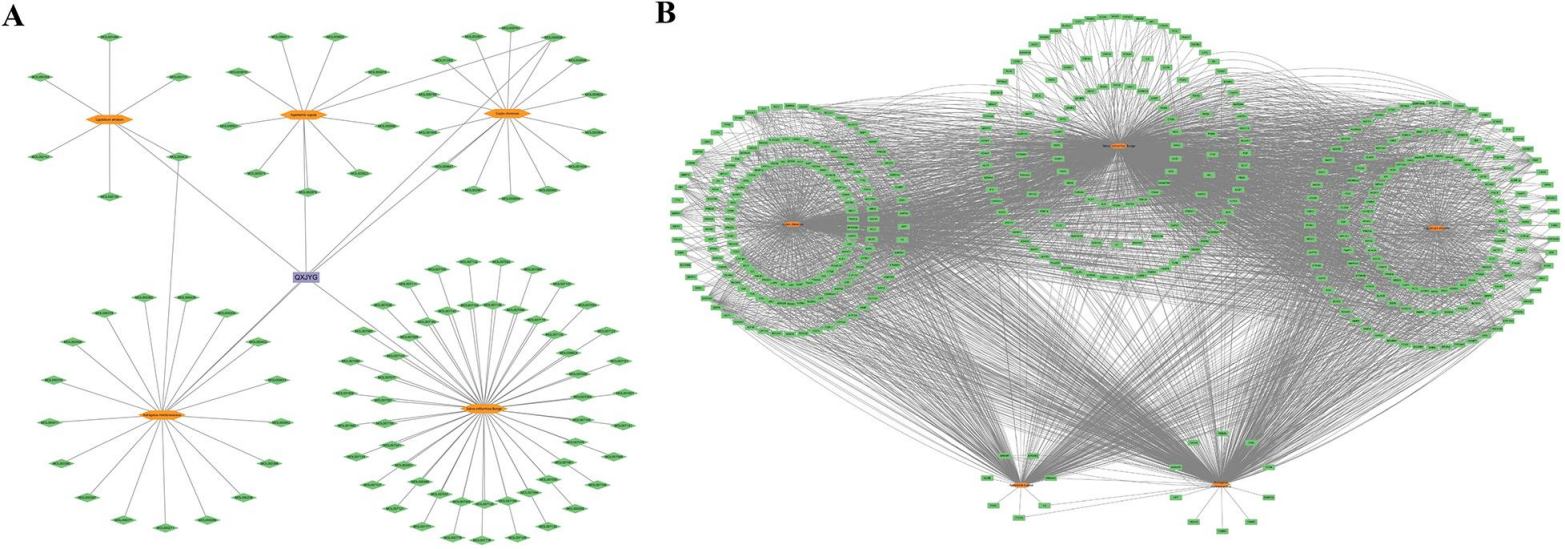
Active ingredients and related targets of QXJYG. **(A)** Active constituents of QXJYG medicinal composition. **(B)** Target genes associated with QXJYG medicinal components. Orange hexagons represent the five medicinal components that make up QXJYG, green diamonds represent the active constituent compounds, green rectangles represent the associated target genes, and purple rectangles represent QXJYG itself.

**Figure 2:**
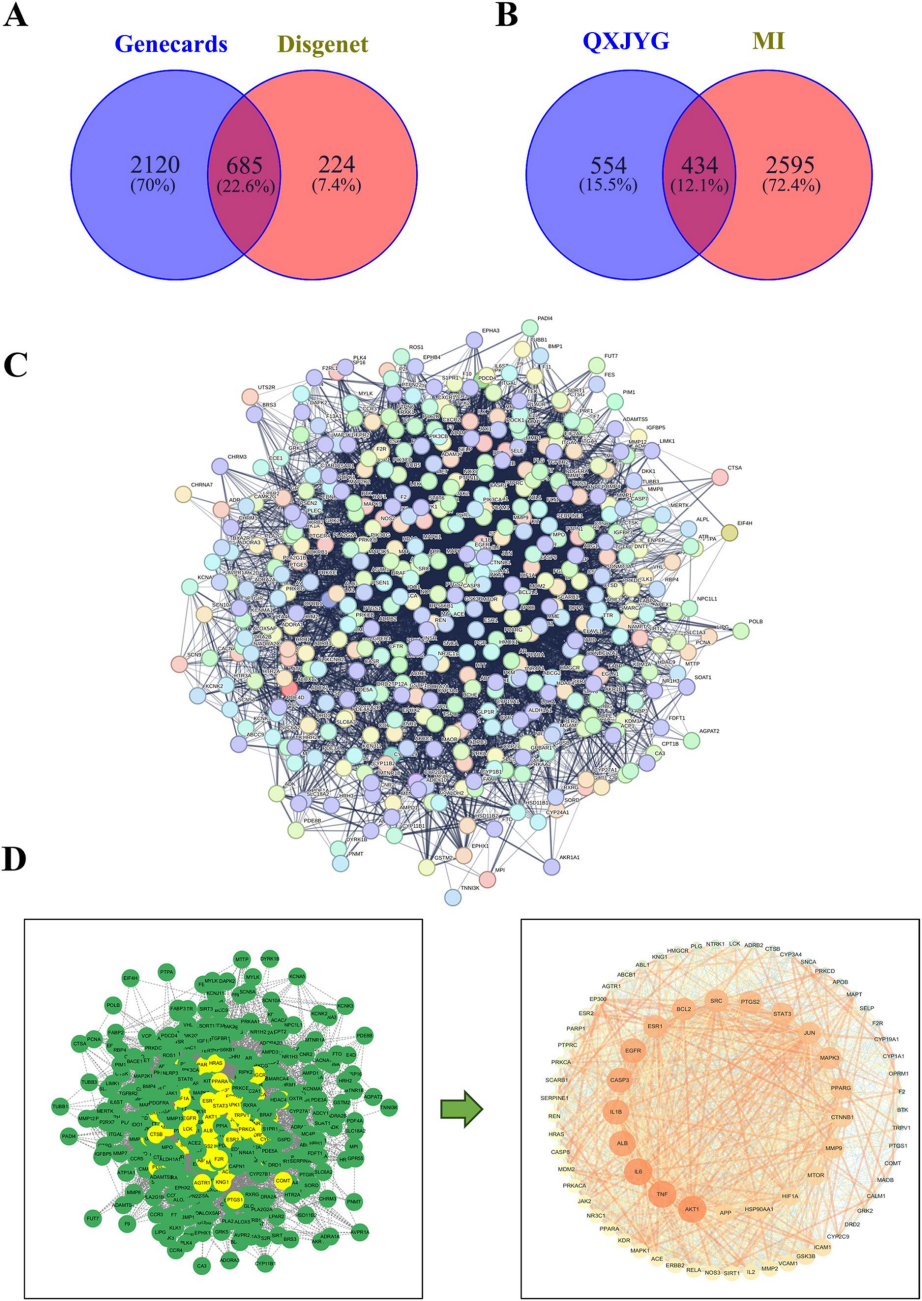
Identification of potential therapeutic targets of QXJYG against MI. **(A)** Screening for MI-related genes using GeneCards and DisGeNET databases. **(B)** Venn diagram of the targets of QXJYG against MI. **(C)** The construction of a protein interaction network of MI target genes induced by QXJYG; **(D)** Screening of core targets of QXJYG in the treatment of MI.

A total of 114 active components and their corresponding 988 targets were collected (Figure 1). A list of the top 20 active components based on OB values was compiled (Table 1). We identified a total of 3,029 MI-related genes from the GeneCards database and the DisGeNET database (Figure 2A). By calculating the intersection of drug targets and disease targets, 434 overlapping targets were identified (Figure 2B). A PPI network was constructed using the STRING database (Figure 2C), and core targets were identified and ranked by degree and betweenness using Cytoscape 3.10.0. The core target network (Figure 2D) suggests that QXJYG may primarily exert its therapeutic effects on MI by regulating these targets, which play crucial roles in various biological processes including inflammation, apoptosis, metabolism, and tissue remodeling.

**Table 1:** The top 20 active ingredients of QXJYG.

Mol ID	Molecule name	OB (%)	DL
MOL007064	Przewalskin B	110.32	0.44
MOL000398	Isoflavanone	109.99	0.3
MOL007132	(2R)-3-(3,4-dihydroxyphenyl)-2-[(Z)-3-(3,4-dihydroxyphenyl)acryloyl]oxy-propionic acid	109.38	0.35
MOL002907	Corchoroside A_qt	104.95	0.78
MOL007140	(Z)-3-[2-[(E)-2-(3,4-dihydroxyphenyl)vinyl]-3,4-dihydroxy-phenyl]acrylic acid	88.54	0.26
MOL008647	Moupinamide	86.71	0.26
MOL007150	(6S)-6-hydroxy-1-methyl-6-methylol-8,9-dihydro-7H-naphtho[8,7-g]benzofuran-10,11-quinone	75.39	0.46
MOL005890	Pachypodol	75.06	0.4
MOL000378	7-O-methylisomucronulatol	74.69	0.3
MOL007058	Formyltanshinone	73.44	0.42
MOL007120	Miltionone Ⅱ	71.03	0.44
MOL000392	Formononetin	69.67	0.21
MOL000433	(2S)-2-[[4-[(2-amino-4-oxo-1H-pteridin-6-yl)methylamino]benzoyl]amino]pentanedioic acid	68.96	0.71
MOL007105	Epidanshenspiroketallactone	68.27	0.31
MOL000438	(3R)-3-(2-hydroxy-3,4-dimethoxyphenyl)chroman-7-ol	67.67	0.26
MOL002140	Perlolyrine	65.95	0.27
MOL007155	Tanshinone IIb	65.26	0.45
MOL000785	Palmatine	64.6	0.65
MOL007130	Prolithospermic acid	64.37	0.31
MOL000380	(6aR,11aR)-9,10-dimethoxy-6a,11a-dihydro-6H-benzofurano[3,2-c] chromen-3-ol	64.26	0.42

### GO and KEGG enrichment analysis of QXJYG against MI

**Figure 3:**
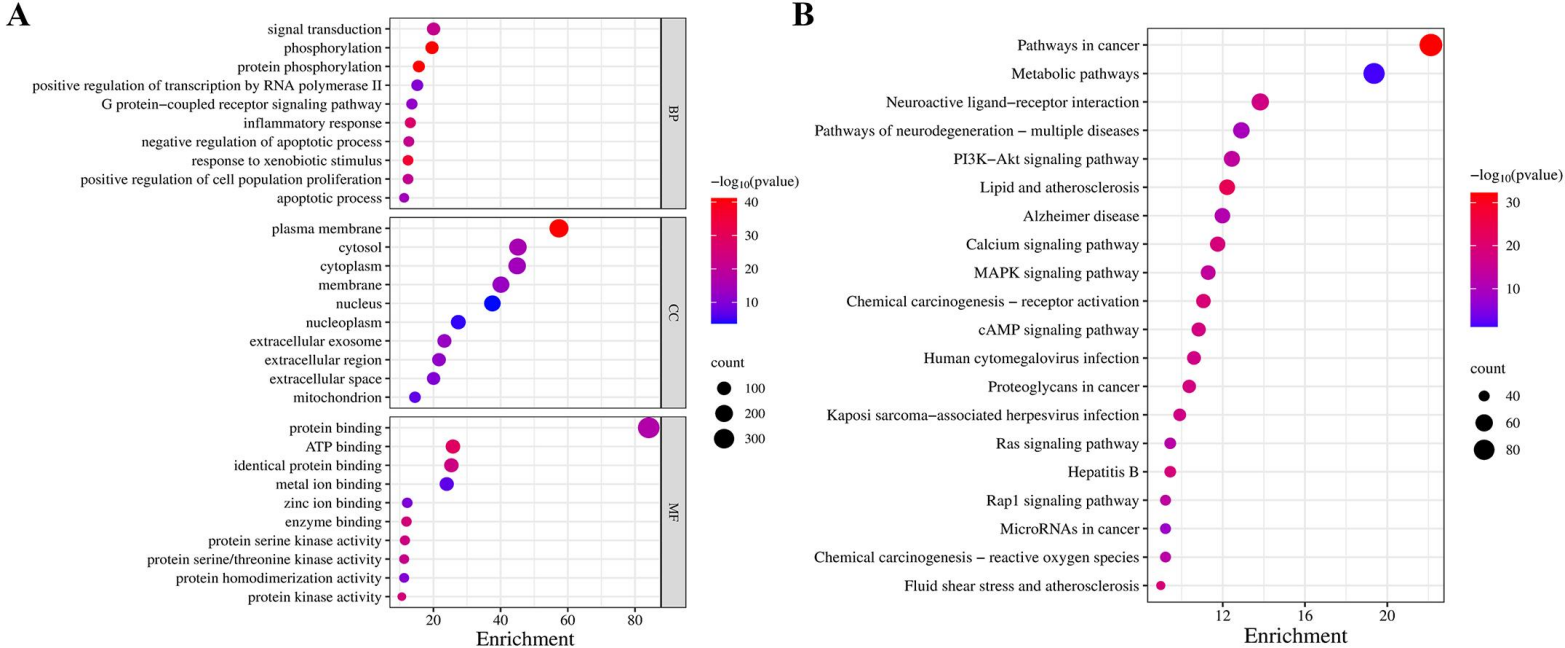
GO and KEGG pathway analysis. **(A)** Top 10 significant GO terms in QXJYG-MI genes (*P* < 0.05). **(B)** Top 20 significantly enriched pathways in KEGG (*P* < 0.05).

To detail the biological characteristics of putative QXJYG targets on MI, GO and pathway enrichment analyses were conducted using the DAVID database. This yielded 1,171 biological processes (BP), 141 cellular components (CC), 291 molecular functions (MF) terms and 196 pathways. Ranked by significance, the top 10 GO terms and the top 20 pathways are shown in Figure 3. QXJYG appears to modulate various biological processes associated with post-MI, including the regulation of protein phosphorylation, which is crucial for signal transduction and cellular response, positive regulation of RNA polymerase II transcription, and the G-protein coupled receptor signaling pathway. Additionally, it may interfere with cell death mechanisms following MI. At the cellular component level, QXJYG is involved in the regulation of extracellular vesicles, cytoplasm, nucleus, nucleoplasm, plasma membrane, and mitochondria post-MI. At the molecular level, QXJYG participates in metal ion binding, zinc ion binding, and enzyme binding, particularly protein serine/threonine kinase activity, indicating its potential to interact with key molecular players in cellular mechanisms. This interaction may influence cellular outcomes post-MI, promoting recovery or adaptation (Figure 3A). Through KEGG pathway enrichment analysis, QXJYG in treating MI potentially regulates multiple critical biological pathways, including lipid metabolism and atherosclerosis, the calcium signaling pathway, the MAPK signaling pathway, the cAMP signaling pathway, and several others. This suggests that QXJYG may have a multifaceted therapeutic effect on MI by modulating various interconnected signaling pathways and biological processes (Figure 3B).

### QXJYG improves cardiac function and pathological changes in MI mice

**Figure 4:**
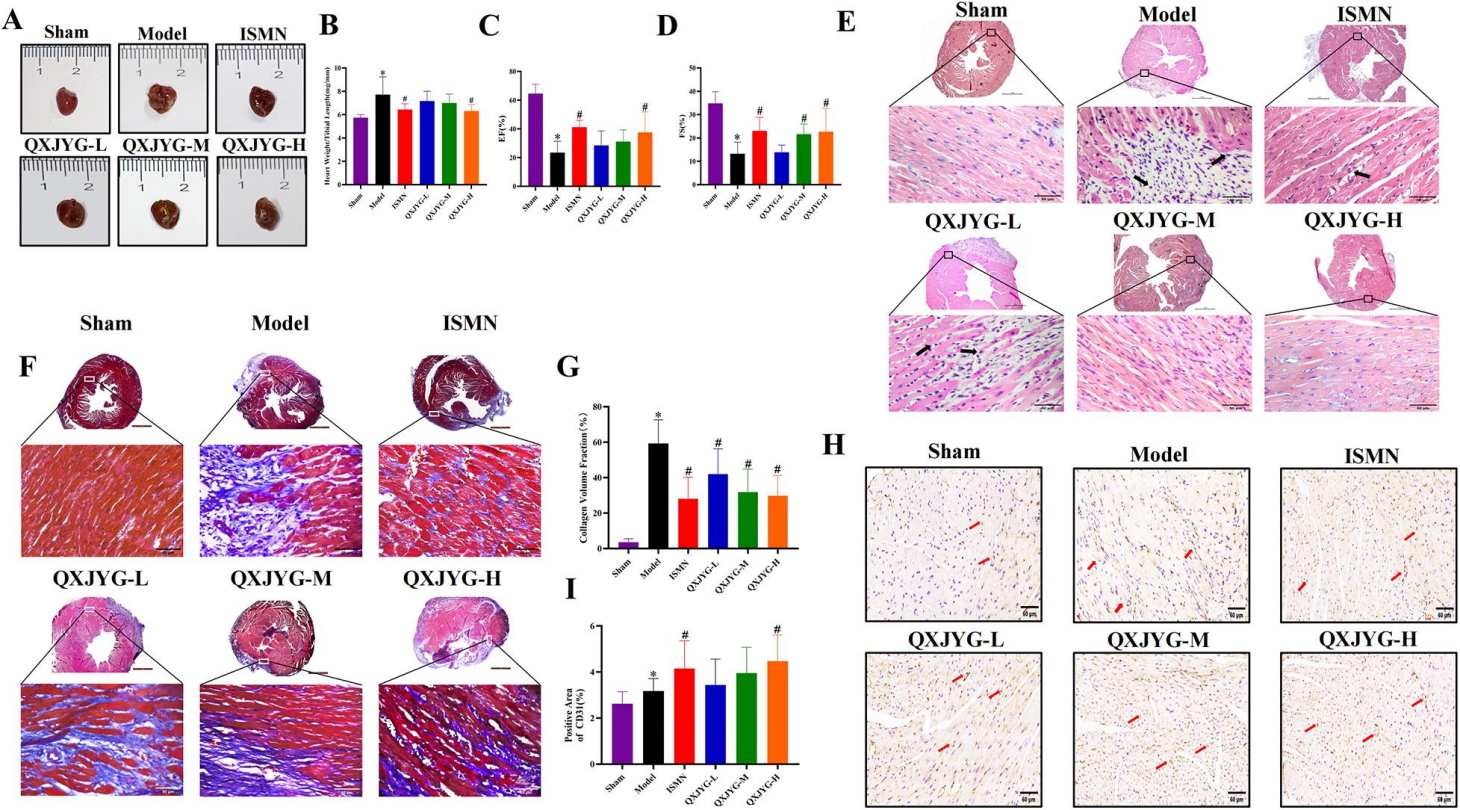
QXJYG improves cardiac function and pathological changes in MI-induced mice. **(A)** Representative graphs of heart morphology in each group; **(B)** The heart weight(mg)/tibial length(mm) was calculated and presented as a histogram (*n* = 6); **(C,D)** The echocardiography measurement of EF, FS was shown (*n* = 6); **(E)** Representative image of hematoxylin and eosin (H&E) staining of each group; Low-magnification images (×25, scale bar = 1 mm) show the overall myocardial morphology, while high-magnification images (×400, scale bar = 50 μm) illustrate local histopathological changes; The area indicated by the black arrow shows inflammatory cell infiltration; **(F,G)** Masson's trichrome–stained histological pictures and quantitation of the area of fibrosis (blue) in cardiac tissues of mice from each group; Low-magnification images (×25, scale bar = 1 mm) present the overall distribution of fibrotic areas, whereas high-magnification images (×400, scale bar=50 μm) show detailed collagen deposition; **(H,I)** Representative image of CD31 (IHC staining) and microvessel density in each group (scale bar = 60 μm). Magnification, ×400. The microvessels marked by the red arrow are CD31-labeled. *P < 0.05 vs. Sham group, ^#^*P* < 0.05 vs. Model group.

Compared to the Sham group, mice in the Model group exhibited significantly enlarged hearts, with the myocardium in the infarcted area of the left ventricular wall appearing pale and depressed, and the ratio of heart weight to tibia length (heart/tibia ratio) was markedly increased (Figures 4A,B, *P < 0.05, vs. Sham Group). Interventions with ISMN and high-dose QXJYG significantly reduced both heart size and the heart/tibia ratio in mice (Figures 4A,B, ^#^*P* < 0.05, vs. Model Group). Echocardiography revealed that both the EF and FS were significantly reduced in the Model group (Figures 4C,D, *P < 0.05, vs. Sham Group), indicating the successful establishment of the MI model. Treatment with ISMN attenuated the reductions in EF and FS observed in the Model group (Figures 4C,D, #*P* < 0.05, vs. Model group). High doses of QXJYG led to significant increases in EF (Figure 4C, ^#^*P* < 0.05, vs. Model group). Moreover, both medium and high doses of QXJYG significantly increased FS (Figure 4D, ^#^*P* < 0.05, vs. Model group).

The HE staining results indicated that in the Sham group, the myocardial cells were organized in a neat spindle shape, with centrally located oval nuclei. The cytoplasm appeared red, and the nuclei were stained purple-blue. Conversely, the Model group displayed thickened myocardial fibers, disorganized cellular arrangement, widened interstitial spaces, and pathological alterations such as vacuolation and infiltration of inflammatory cells within the interstitium (as indicated by arrows). These pathological changes showed improvement following intervention with low, medium, and high doses of QXJYG and ISMN (Figure 4E). Masson's trichrome staining revealed that in the Sham group, myocardial cells were arranged in an orderly fashion with minimal collagen fiber deposition, which did not significantly disrupt myocardial structure. In contrast, the Model group exhibited substantial collagen fiber deposition, irregular myocardial cell arrangement, and structural disarray (Figures 4F,G, *P < 0.05, vs. Sham Group). Following intervention with ISMN and low, medium, and high doses of QXJYG, there was a notable reduction in collagen fiber deposition in cardiac tissue and an improvement in myocardial structure (Figures 4F,G, ^#^*P* < 0.05, vs. Model Group). Quantitative assessment of mouse cardiac microvascular density (MVD) was conducted using immunohistochemistry with specific antibodies against CD31 antigen to visualize the cardiac microvascular network. Compared to the Sham group, the Model group exhibited a significant increase in MVD (Figures 4H,I, *P < 0.05, vs. Sham Group). Furthermore, compared to the Model group, both the ISMN group and the high-dose QXJYG group showed a significant increase in MVD ((Figures 4H,I, ^#^*P* < 0.05, vs. Model Group).

These experimental findings indicate that QXJYG treatment effectively mitigates cardiac enlargement, enhances myocardial structure, and reduces collagen deposition in a mouse model of MI, thereby preserving cardiac function.

### QXJYG mitigates oxidative stress and apoptosis in MI-induced mice via p38 MAPK signaling pathway

**Figure 5:**
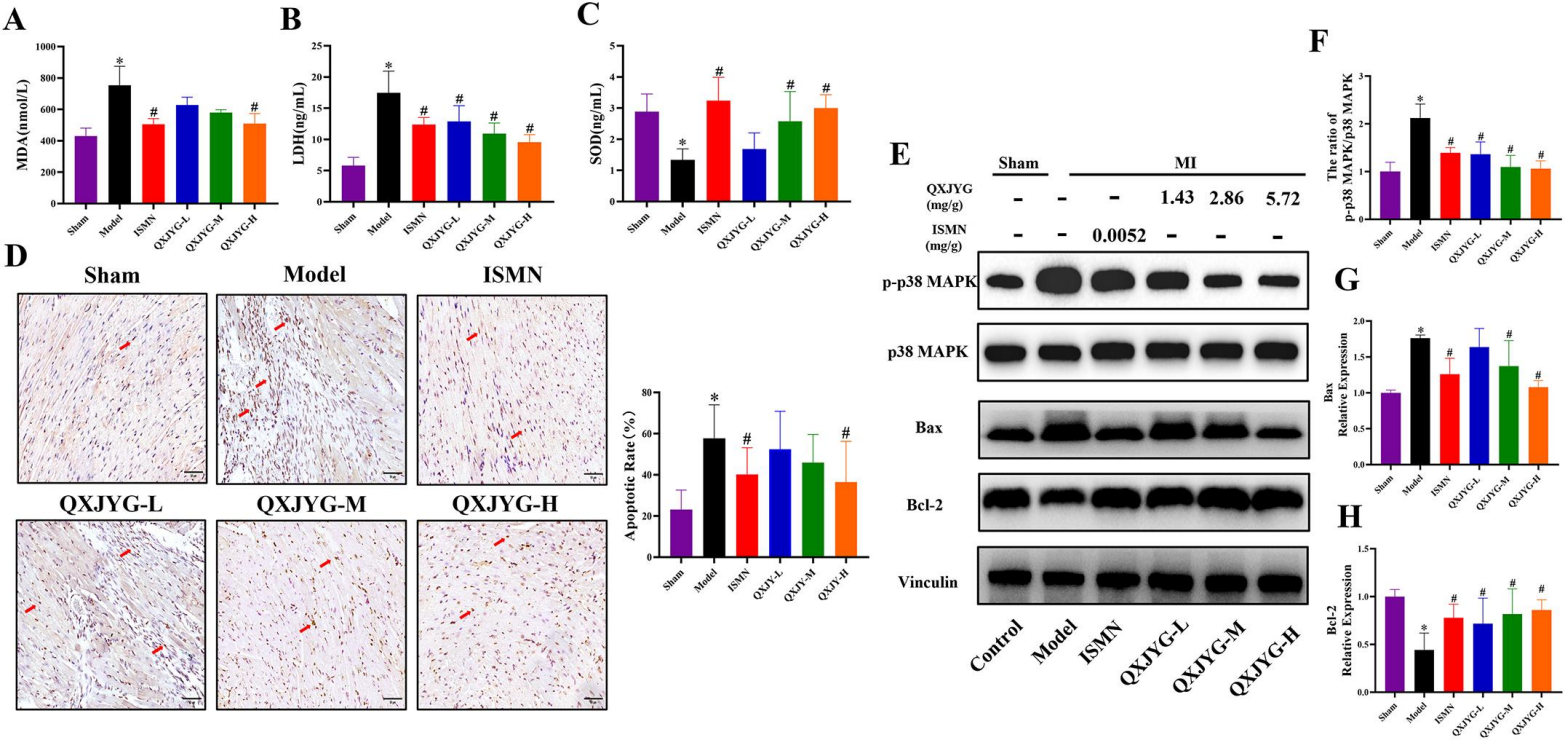
QXJYG mitigates oxidative stress and apoptosis in MI-induced mice via p38 MAPK signaling pathway **(A)** MDA levels in the serum of each group; **(B)** LDH levels in the serum of each group; **(C)** SOD levels in the serum of each group; **(D)** representative image of TUNEL staining and quantification of the apoptotic cells (brown) in cardiac tissues of mice from each group (scale bar = 50 μm); the area indicated by the red arrow shows apoptotic cells; magnification, ×400; **(E–H)** the protein expression of p-p38 MAPK, p38 MAPK, Bax, Bcl-2; Vinculin was used as the internal control. Data are presented as mean±*SD* from three mice per group (*n* = 3). *P < 0.05 vs. Sham group, ^#^*P* < 0.05 vs. Model group.

The levels of MDA and LDH in the serum of mice were found to be significantly increased in the Model group (Figures 5A,B, *P < 0.05, vs. Sham group), whereas the SOD level was decreased (Figure 5C, *P < 0.05, vs. Sham group). The MI-induced mice treated with ISMN and high-dose QXJYG showed significantly reduced MDA levels (Figure 5A, ^#^*P* < 0.05, vs. Model group). LDH levels exhibited a significant reduction in the ISMN group and in all dose groups of QXJYG (Figure 5B, ^#^*P* < 0.05, vs. Model group). Serum SOD levels were significantly increased in the ISMN and QXJYG-M, QXJYG-H groups (Figure 5C, ^#^*P* < 0.05, vs. Model group).

We further determined the effect of QXJYG on cardiomyocyte apoptosis in MI-induced mice using TUNEL staining. The results revealed that compared to the Sham group, the Model group exhibited a significant increase in the rate of apoptotic cells (Figure 5D, *P < 0.05, vs. Sham group). Conversely, mice treated with ISMN and high-dose QXJYG showed a significant reduction in the rate of cell apoptosis in cardiac tissue (Figure 5D, ^#^*P* < 0.05, vs. Model group). Western blot analysis was then used to detect the expression of apoptosis-related proteins. The results showed that the protein expression of pro-apoptotic Bax in the cardiac tissue of the Model group were significantly increased (Figures 5E,G, *P < 0.05, vs. Sham group), while the protein expression of anti-apoptotic Bcl-2 was significantly decreased (Figures 5E, H, *P < 0.05, vs. Sham group). The protein expression of Bax was significantly decreased in the ISMN, QXJYG-M, and QXJYG-H groups compared to the Model group (Figures 5E,G, ^#^*P* < 0.05, vs. Model group). Additionally, the protein expression of Bcl-2 was significantly increased in the ISMN, QXJYG-L, QXJYG-M, and QXJYG-H groups compared to the Model group (Figures 5E,H, ^#^*P* < 0.05, vs. Model group).

Emerging evidence has shown that the activated p38 MAPK signaling pathway is involved in oxidative stress and apoptosis during the progression of MI (13, 14). Our findings displayed that the ratio of p-p38 MAPK/p38 MAPK was significantly increased in the cardiac tissue of MI-induced mice (Figures 5E,F, *P < 0.05, vs. Sham group). However, there was a decrease in the ratio of p-p38 MAPK/p38 MAPK in the ISMN and QXJYG-L, QXJYG-M, and QXJYG-H groups in comparison to the Model group (Figure 5E,F, ^#^*P* < 0.05, vs. Model group). Taken together, our results indicated that QXJYG inhibited oxidative stress and apoptosis in cardiac tissue of MI-induced mice via suppression of the p38 MAPK signaling pathway.

### QXJYG reduces hypoxia-induced oxidative stress and apoptosis in H9C2 cells via p38 MAPK signaling pathway

**Figure 6:**
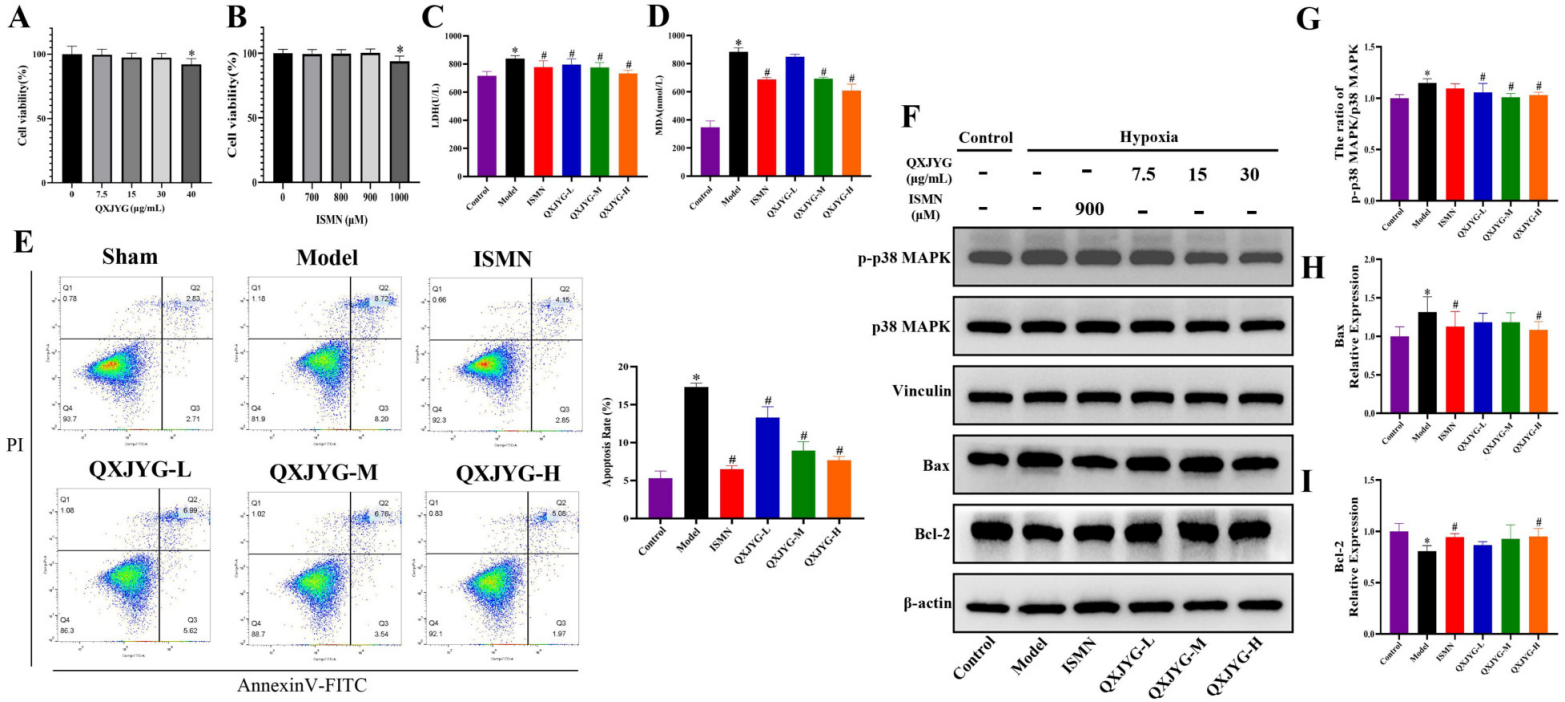
QXJYG reduces hypoxia-induced oxidative stress and apoptosis in H9C2 cells via p38 MAPK signaling pathway. **(A)** CCK-8 assay for screening the optimal concentration of QXJYG intervention in H9C2 Cells; **(B)** CCK-8 assay for screening the optimal concentration of ISMN intervention in H9C2 Cells; **(C)** LDH levels in the cell culture supernatants of each group; **(D)** MDA levels in the cell culture supernatants of each group; **(E)** Representative flow cytometry images and apoptotic cell quantification bar graphs of H9C2 cells in each group; **(F–I)** The protein expression of p-p38 MAPK, p38 MAPK, Bax, Bcl-2; Vinculin and β-actin was used as the internal control. Data are presented as mean±*SD* from three independent biological replicates (*n* = 3). *P < 0.05 vs. Sham group, ^#^*P* < 0.05 vs. Model group.

To further elucidate the efficacy and mechanism of QXJYG against MI, we established an *in vitro* model of hypoxia-induced H9C2 cells. To determine the optimal concentrations of QXJYG and ISMN for subsequent *in vitro* experiments, we first performed a dose-response analysis using the CCK-8 assay. H9C2 cells were treated with QXJYG at concentrations of 0, 7.5, 15, 30, and 40 μg/mL for 24 h. Cell viability remained above 85% and showed no significant difference compared to the control group at 7.5, 15, and 30 μg/mL (*P* > 0.05), indicating that these concentrations were non-cytotoxic. However, a significant reduction in viability was observed at 40 μg/mL (Figure 6A, *P < 0.05, vs. Control group), suggesting the onset of cytotoxicity. Therefore, 7.5, 15, and 30 μg/mL were selected as the low, medium, and high concentrations, respectively, for subsequent experiments. Similarly, ISMN was tested at concentrations of 0, 700, 800, 900, and 1,000 μM. ISMN at 700–900 μM did not significantly affect cell viability compared to the control group, while 1,000 μM led to a significant reduction in viability, indicating cytotoxicity at this concentration. Therefore, 900 μM was selected as the working concentration for subsequent *in vitro* experiments due to its favorable safety profile and stable biological activity (Figure 6B, *P < 0.05, vs. Control group).

Secondly, the detection results of MDA and LDH levels in cell culture supernatant showed that the levels of MDA and LDH in the Model group were significantly increased (Figures 6C,D, *P < 0.05, vs. Control group). Compared to the Model group, LDH levels of hypoxia-induced H9C2 cells significantly were decreased following intervention with ISMN and low, medium, and high doses of QXJYG (Figure 6C, ^#^*P* < 0.05, vs. Model group). Simultaneously, treatment with ISMN, as well as medium and high doses of QXJYG, substantially reduced MDA levels (Figure 6D, ^#^*P* < 0.05, vs. Model group).

Thirdly, the cell apoptosis was determined via Annexin V/PI staining followed by FACS analysis. The results revealed a significant increase in the rate of apoptotic cells in the Model group (Figure 6E, *P < 0.05, vs. Control group). Conversely, cells treated with ISMN and low, medium, and high doses of QXJYG showed a significant reduction in apoptosis (Figure 6E, ^#^*P* < 0.05, vs. Model group).

We further used western blot analysis to evaluate whether the inhibitory effect of QXJYG on oxidative stress and apoptosis via regulating the p38 MAPK signaling pathway in hypoxia-induced H9C2 cells. The results showed that the ratio of p-p38 MAPK/p38 MAPK and the protein expression of Bax were significantly increased in the hypoxia-induced H9C2 cells (Figures 6F–H, *P < 0.05, vs. Control group), while the protein expression of Bcl-2 was significantly decreased (Figures 6F,I, *P < 0.05, vs. Control group). The ratio of p-p38 MAPK/p38 MAPK was significantly decreased in the QXJYG-L, QXJYG-M, and QXJYG-H groups compared to the Model group (Figures 6F,G, ^#^*P* < 0.05, vs. Model group). Additionally, Bax protein expression showed a significant reduction in the ISMN and QXJYG-H groups relative to the Model group (Figure 6F,H, ^#^*P* < 0.05, vs. Model group). In contrast, the protein expression of Bcl-2 was markedly increased in the ISMN, and QXJYG-H groups when compared to the Model group (Figures 6F,I, ^#^*P* < 0.05, vs. Model group). Collectively, we conclude that QXJYG alleviated the hypoxia-induced oxidative stress and apoptosis through downregulation of p38 MAPK signaling pathway.

### The combination of QXJYG and SB203580 synergistically attenuates hypoxia-induced injury via p38 MAPK inhibition

**Figure 7:**
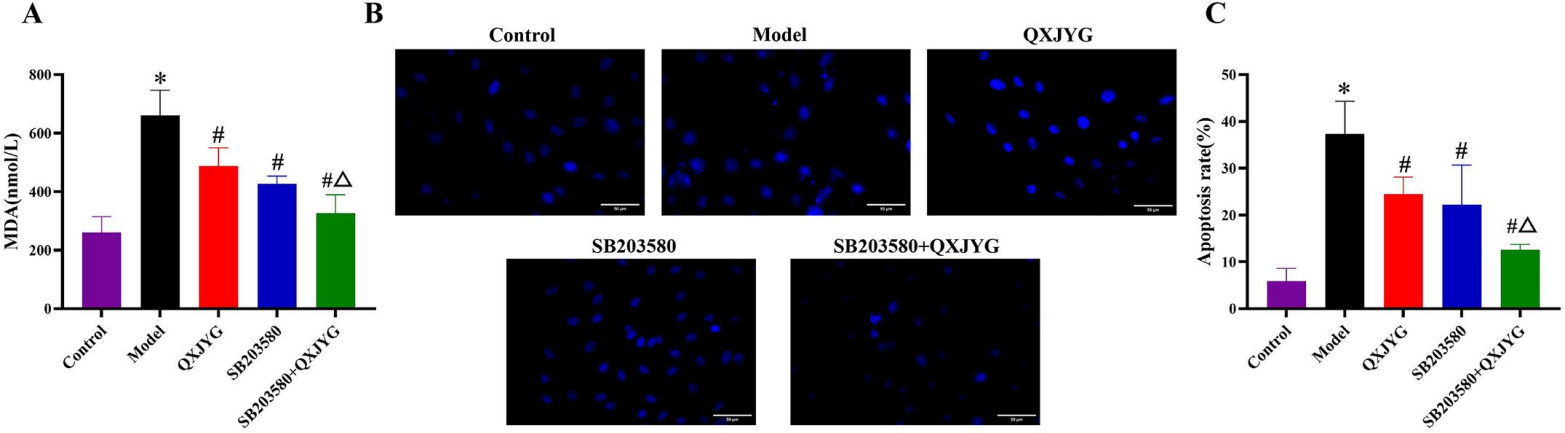
QXJYG reduces hypoxia-induced MDA accumulation and apoptosis via p38 MAPK inhibition. **(A)** MDA levels in the cell culture supernatants of each group. **(B)** Representative images of Hoechst 33,258 staining in the different groups. Magnification, ×400, Scale bar = 50 μm. **(C)** Apoptosis rate of H9C2 cells; *P < 0.05 vs. Control; ^#^*P* < 0.05 vs. Model; ^Δ^*P* < 0.05 vs. QXJYG.

To further investigate the mechanism, SB203580, a p38 MAPK inhibitor, and its combination with high-dose QXJYG were used. Both treatments significantly decreased MDA levels compared to the Model group (Figure 7A, #*P* < 0.05, vs. Model group), with a more pronounced reduction observed in the combined treatment group compared to QXJYG treatment alone (Figure 7A, ^Δ^*P* < 0.05, vs. QXJYG group), suggesting a potential synergistic antioxidative effect. Hoechst 33,258 staining further revealed a significant increase in apoptotic nuclei in hypoxia-induced H9C2 cells compared to the Control group (Figures 7B,C, *P < 0.05 vs. Control group). Treatment with QXJYG markedly reduced the number of apoptotic cells compared to the hypoxia group (Figures 7B,C, ^#^*P <* 0.05 vs. Model group), indicating its protective effect against hypoxia-induced apoptosis. Notably, pretreatment with the p38 MAPK inhibitor SB203580 also significantly decreased apoptosis levels (Figures 7B,C, ^#^*P <* 0.05 vs. Model group). Furthermore, combined treatment with SB203580 and QXJYG resulted in an even greater reduction in apoptotic cells (Figures 7B,C, ^Δ^*P <* 0.05 vs. QXJYG group). These findings offer further mechanistic support for the involvement of the p38 MAPK signaling pathway in mediating the anti-apoptotic effects of QXJYG under hypoxic conditions.

## Discussion

From the perspective of TCM, QXJYG was originally developed and clinically applied for patients with CHD, particularly those with stable coronary artery disease (CAD) and angina pectoris. Within the TCM framework, these conditions are discussed under the syndromic categories of “Xiong Bi” and “Zhen Xin Tong”, which represent symptom-based classifications characterized by recurrent chest pain, chest tightness, and exertional discomfort. Based on long-term clinical practice, QXJYG has been widely used to alleviate angina symptoms, improve exercise tolerance, and reduce inflammatory activity in patients with stable CAD (15–17).

Although MI is not explicitly described in classical TCM texts, its acute clinical manifestations share substantial similarities with severe stages of these syndromic presentations, particularly in terms of abrupt exacerbation of chest pain, circulatory disturbance, and rapid deterioration of cardiac function. Importantly, TCM emphasizes disease progression rather than isolated disease entities. Stable CAD and MI can be viewed as different stages along a shared pathological continuum, in which prolonged functional impairment and circulatory disturbance may progress toward more severe states associated with intensified inflammation, endothelial dysfunction, and acute coronary perfusion impairment.

Within this theoretical framework, QXJYG was developed to target key pathological features of CAD characterized by blood stasis and toxin accumulation, with the therapeutic goal of modulating inflammatory activity and vascular dysfunction during the stable stage of the disease. Previous clinical studies have demonstrated that QXJYG significantly improves inflammatory biomarkers, endothelial function, and clinical outcomes in patients with stable CAD, indicating its potential role in reducing plaque instability and systemic inflammatory burden (4, 18, 19). As MI arises from plaque instability and acute coronary occlusion, these established clinical effects provide a pathophysiological rationale for further investigating the protective role of QXJYG in MI. Consistent with the established clinical benefits of QXJYG in stable CAD, the present study demonstrates that QXJYG significantly ameliorates post-infarction cardiac dysfunction and adverse myocardial remodeling, as evidenced by improvements in left ventricular systolic performance, attenuation of myocardial structural injury, suppression of inflammatory infiltration and fibrotic deposition, and preservation of microvascular integrity. These integrated pharmacodynamic effects provide a critical pathological and functional basis for further mechanistic investigations in the post-infarction myocardium.

After MI, ischemia and hypoxia in myocardial tissue reduce the efficiency of the mitochondrial electron transport chain, leading to excessive production of reactive oxygen species such as superoxide anions, which is exacerbated by cell death and metabolic disruption that release additional intracellular reactive oxygen species (ROS) (20, 21). This process exacerbates damage to myocardial cells, impacting cardiac function and impairing the repair process. SOD, a critical component of the antioxidant defense system, mitigates oxidative damage by scavenging superoxide anions (22). Experimental findings demonstrated that QXJYG increased SOD levels of serum in MI-induced mice. MDA levels, typically elevated following MI due to oxidative stress-induced membrane damage, were significantly reduced in the serum of MI-induced mice following QXJYG treatment. Moreover, results from *in vitro* experiments revealed that QXJYG treatment also significantly reduced MDA levels in hypoxia-induced H9C2 cells. LDH, an enzyme involved in cellular metabolism, particularly in the conversion between lactate and pyruvate, reflects cell membrane damage and death (23). QXJYG effectively reduced LDH levels in both the serum of the MI-induced mice and the supernatant of hypoxia-induced H9C2 cells, thereby protecting against cellular injury. The convergence of *in vivo* and *in vitro* experiments confirms that QXJYG alleviates oxidative stress post-MI and mitigates myocardial cell damage.

Oxidative stress following MI can induce cardiomyocyte apoptosis through multiple pathways, such as disrupting mitochondrial permeability and function, as well as through endoplasmic reticulum stress and death receptor signaling (24, 25). As a form of programmed cell death, the activation of apoptosis after MI further worsens cardiac function and structure, ultimately compromising overall heart health (26). Through GO enrichment analysis, we found that QXJYG may regulate the apoptosis of cardiomyocytes post-MI by targeting mitochondrial components. In the mitochondrial apoptosis pathway, Bcl-2 family proteins play crucial roles in regulating mitochondrial outer membrane permeability (MOMP) (27). Ischemia and hypoxia increase intracellular calcium ions and oxidative stress, activating pro-apoptotic proteins such as Bax, which form pores in the mitochondrial outer membrane, thereby increasing permeability and releasing apoptotic factors into the cytoplasm, initiating apoptosis (28). Conversely, anti-apoptotic proteins like Bcl-2 competitively bind and inhibit Bax, maintaining mitochondrial integrity and preventing pore formation, thereby protecting cardiomyocytes from apoptosis (29). A higher Bax/Bcl-2 ratio, caused by aberrant expression of the proteins, is often seen after MI. TUNEL staining showed significantly reduced apoptosis in cardiomyocytes of MI-induced mice treated with high-dose QXJYG. Furthermore, Annexin V/PI staining demonstrated QXJYG significantly reduced hypoxia-induced apoptosis in H9C2 cells. At the protein expression level, QXJYG intervention increased Bcl-2 protein expression and decreased Bax protein expression in both heart tissues of MI-induced mice and hypoxia-induced H9C2 cells. These experimental findings are consistent with the findings from network pharmacology analyses, confirming the ability of QXJYG to protect the heart by regulating apoptosis in cardiomyocytes post-MI.

MAPKs are highly conserved serine/threonine protein kinases that transmit extracellular signals into cells via a three-tiered kinase cascade, playing a critical role in regulating cell growth, differentiation, proliferation, and apoptosis (30). Previous studies have revealed that the p38 MAPK signaling pathway can be triggered and activated during acute MI injury by changes in cellular osmotic pressure, decreased levels of energy metabolites such as ATP and creatine phosphate, and activation of inflammatory cytokines (31–35). The experimental study indicates that inhibiting the activation of p38 MAPK in the hypoxia-reoxygenation cardiomyocyte model can alleviate the oxidative stress response in cells (13). Furthermore, activated p38 MAPK can enhance apoptosis of cardiomyocytes through mechanisms such as increasing the expression of C-MYC protein, phosphorylating tumor suppressor protein p53, activating transcription factors c-Jun and c-Fos, and inducing pro-apoptotic protein Bax translocation from the cytoplasm to mitochondria, exacerbating myocardial injury and dysfunction (36–39). GO and KEGG enrichment analyses indicated that QXJYG may improve cardiac function after MI by modulating protein kinase activity and kinase activity, particularly through the MAPK pathway (hsa04010).

Previous studies have demonstrated that Astragalus polysaccharide (APS) attenuates hypoxia/reoxygenation-induced injury in human cardiac microvascular endothelial cells (HCMECs) by suppressing the phosphorylation of p38 MAPK and downregulating adhesion molecule expression (40). Astragaloside IV (AS-IV), another major component of *Astragalus membranaceus*, has been shown to reduce p38 MAPK phosphorylation, inhibit myocardial fibrosis, and improve left ventricular remodeling in a mouse model of dilated cardiomyopathy (41). Similarly, *Salvia miltiorrhiza* extract significantly decrease myocardial infarct size in rat models of myocardial ischemia, potentially via inhibition of p38 MAPK activation (42). Given that *Astragalus membranaceus* and *Salvia miltiorrhiza* are key constituents of QXJYG, these findings provide a pharmacological basis supporting the hypothesis that QXJYG may exert cardioprotective effects by suppressing p38 MAPK phosphorylation. Furthermore, our study identified several key active constituents of QXJYG with potential activity against the p38 MAPK pathway. Palmatine has been reported to suppress LPS-induced pro-inflammatory cytokine production by downregulating the phosphorylation of p38 MAPK (43). Similarly, isoflavanone effectively inhibits p38 MAPK phosphorylation, thereby preventing the activation of downstream pro-apoptotic proteins such as Bax (44). Moreover, isoflavanone dose-dependently suppresses the phosphorylation of p38 and other MAPK family members, collectively contributing to the attenuation of inflammatory and apoptotic signaling pathways (45). In addition, molecular docking studies reveals that pachypodol and 7-O-methylisomucronulatol exhibit high binding affinity toward p38 MAPK, suggesting their potential as direct modulators of this signaling cascade (46, 47). These results collectively suggest that QXJYG may alleviate myocardial ischemia and hypoxia-induced cardiomyocyte injury, through inhibition of the p38 MAPK signaling pathway.

Consistent with this prediction, *in vivo* and *in vitro* western blot analyses confirmed that QXJYG significantly reduced the phosphorylation level of p38 MAPK in both MI-induced mouse heart tissue and hypoxia-treated H9C2 cells, indicating effective inhibition of the pathway. To further validate the mechanistic involvement of p38 MAPK, we conducted additional *in vitro* experiments using the specific p38 MAPK inhibitor SB203580. Our results revealed that both SB203580 and QXJYG significantly reduced MDA levels in hypoxia-induced H9C2 cells. Importantly, their combination exerted a more pronounced inhibitory effect on MDA accumulation compared to QXJYG treatment alone. Hoechst 33,258 staining revealed that both SB203580 and QXJYG independently attenuated hypoxia-induced apoptosis in H9C2 cells. Moreover, the combination of QXJYG and SB203580 further reduced the apoptotic rate compared with QXJYG treatment alone. These findings provide direct functional evidence that the anti-oxidant and anti-apoptotic effect of QXJYG is at least partially mediated through inhibition of the p38 MAPK signaling pathway. However, the precise molecular interactions between QXJYG's multiple components and biological targets require further elucidation. Future research should focus on elucidating these interactions and optimizing the formulation to enhance its efficacy in treating MI and related complications.

## Conclusion

In summary, using network pharmacology and experimental validation, we explored the therapeutic mechanisms of QXJYG in MI. We found that QXJYG exerts cardioprotective effects against MI by improving cardiac function, reducing oxidative stress, and inhibiting apoptosis through the regulation of the p38 MAPK signaling pathway both *in vivo* and *in vitro*.

## Data Availability

The original contributions presented in the study are included in the article, further inquiries can be directed to the corresponding authors.

## References

[1] Solomon SD Skali H Anavekar NS Bourgoun M Barvik S Ghali JK Changes in ventricular size and function in patients treated with valsartan, captopril, or both after myocardial infarction. Circulation. (2005) 111(25):3411–9. 10.1161/circulationaha.104.508093 15967846

[2] Lee TH Hamilton MA Stevenson LW Moriguchi JD Fonarow GC Child JS Impact of left ventricular cavity size on survival in advanced heart failure. Am J Cardiol. (1993) 72(9):672–6. 10.1016/0002-9149(93)90883-e 8249843

[3] Xu H Shi D Yin H Zhang J Chen K. Blood-stasis and toxin causing catastrophe hypothesis and acute cardiovascular events: proposal of the hypothesis and its clinical significance. Chin J Integr Tradit West Med. (2008) 10:934–8. 19123336

[4] Li J Gao Z Zhang L Li S Yang Q Shang Q Qing-Xin-Jie-Yu granule for patients with stable coronary artery disease (quest trial): a multicenter, double-blinded, randomized trial. Complement Ther Med. (2019) 47:102209. 10.1016/j.ctim.2019.102209 31780034

[5] Li J. A randomised double-blind controlled study of the effect of qing-Xin-jie-yu fang on clinical endpoint events in stable coronary heart disease (Dissertation/doctor’s thesis). Beijing University of Chinese Medicine, Beijing (2018).

[6] Ju J. Mechanism study on stabilisation of atherosclerotic vulnerable plaques by regulating macrophage pyroptosis with qing-Xin-jie-yu fang prescription (Dissertation/doctor’s thesis). Beijing University of Traditional Chinese Medicine, Beijing (2019).

[7] Gao X. Clicinal study of qing-xin-jie-yu decoction on elevated serum levels of inflammatory markers in patients with stable coronary heart disease (dissertation/master's thesis). Chinese Academy of Chinese Medicine, Beijing (2017).

[8] Wang A Guan B Shao C Zhao L Li Q Hao H Qing-Xin-Jie-Yu granule alleviates atherosclerosis by reshaping gut microbiota and metabolic homeostasis of apoe^−/−^ mice. Phytomedicine. (2022) 103:154220. 10.1016/j.phymed.2022.154220 35675748

[9] Qi M Huang H Li Z Quan J Wang J Huang F Qingxin Jieyu Granule alleviates myocardial infarction through inhibiting neutrophil extracellular traps via activating Anxa1/Fpr2 axis. Phytomedicine. (2024) 135:156147. 10.1016/j.phymed.2024.156147 39418972

[10] Li M Chen S Wang X Jiang Z Li L Xu Y Study on determination of multi-index components and uplc fingerprint of standard decoction of Qingxin Jieyu formula. J Nanjing Univ Tradit Chin Med. (2021) 37(03):419–27. 10.14148/j.issn.1672-0482.2021.0419

[11] Jiang K Chen Y. Mechanism of mir-101-3p protecting myocardial ischemia-reperfusion injury through MAPK signaling pathway. Mod Med J. (2022) 50(06):665–71. 10.3969/j.issn.1671-7562.2022.06.001

[12] Shen Z Shen A Chen X Wu X Chu J Cheng Y Huoxin pill attenuates myocardial infarction-induced apoptosis and fibrosis via suppression of P53 and Tgf-*Β*1/Smad2/3 pathways. Biomed Pharmacother. (2020) 130:110618. 10.1016/j.biopha.2020.110618 34321167

[13] Ashraf MI Ebner M Wallner C Haller M Khalid S Schwelberger H A p38 MAPK/Mk2 signaling pathway leading to redox stress, cell death and ischemia/reperfusion injury. Cell Commun Signal. (2014) 12:6. 10.1186/1478-811x-12-6 24423080 PMC3896752

[14] Chang NC Yeh CT Lin YK Kuo KT Fong IH Kounis NG Garcinol attenuates lipoprotein(a)-induced oxidative stress and inflammatory cytokine production in ventricular cardiomyocyte through *α*7-nicotinic acetylcholine receptor-mediated inhibition of the P38 MAPK and Nf-*Κ*b signaling pathways. Antioxidants. (2021) 10(3):461. 10.3390/antiox10030461 33809417 PMC8000018

[15] Shi L Gao Z Li J Xue M Song L Li S Effect of qingxin jieyu formula on the long-term prognosis of patients with stable coronary artery disease:a prospective cohort study. Chin J Integr Tradit West Med. (2022) 42(11):1293–9. 10.7661/j.cjim.20220914.285

[16] Zhou X Zhong Z Wang Y Zhao Q Zhao J Ma T. Effect of Qingxin Jieyu recipe on stable coronary heart disease and changes of keratin 6B, 16 and a in patients. Chin Arch Tradit Chin Med. (2019) 37(12):3065–9. 10.13193/j.issn.1673-7717.2019.12.060

[17] Chen L Dai L Xu J Duan L Hou X Zhang L Chinese herbal compound preparation Qing-Xin-Jie-Yu Granules for intermediate coronary lesions in patients with stable coronary artery disease: study protocol for a multicenter, randomized, double-blind, placebo-controlled trial. PLoS One. (2024) 19(7):e0307074. 10.1371/journal.pone.0307074 39012918 PMC11251585

[18] Li S Guo M Mao H Gao Z Xu H Shi D. Qing-Xin-Jie-Yu granules in addition to conventional treatment for patients with stable coronary artery disease (Quest Trial): study protocol for a randomized controlled trial. Trials. (2016) 17(1):451. 10.1186/s13063-016-1569-9 27628038 PMC5024507

[19] Ouyang J Shi J Jiang Z Qu H Zhang L Hu Z Qingxin jieyu granule for the treatment of acutecoronary syndrome with binding of stasis and toxin syndrome:a multicenter,double-blind,placebo randomize-controlled trial. J Tradit Chin Med. (2025) 66(22):2345–52. 10.13288/j.11-2166/r.2025.22.010

[20] Cai S Zhao M Zhou B Yoshii A Bugg D Villet O Mitochondrial dysfunction in macrophages promotes inflammation and suppresses repair after myocardial infarction. J Clin Invest. (2023) 133(4):e159498. 10.1172/jci159498 36480284 PMC9927948

[21] Jin S Kang PM. A systematic review on advances in management of oxidative stress-associated cardiovascular diseases. Antioxidants. (2024) 13(8):923. 10.3390/antiox13080923 39199169 PMC11351257

[22] Zhong Y Yang Y Xu Y Qian B Huang S Long Q Design of a zn-based nanozyme injectable multifunctional hydrogel with ros scavenging activity for myocardial infarction therapy. Acta Biomater. (2024) 177:62–76. 10.1016/j.actbio.2024.01.015 38237713

[23] Shahzad S Mateen S Mubeena Mariyath PM Naeem SS Akhtar K Rizvi W Protective effect of syringaldehyde on biomolecular oxidation, inflammation and histopathological alterations in isoproterenol induced cardiotoxicity in rats. Biomed Pharmacother. (2018) 108:625–33. 10.1016/j.biopha.2018.09.055 30245462

[24] Wang Y Li Q Zhao J Chen J Wu D Zheng Y Mechanically induced pyroptosis enhances cardiosphere oxidative stress resistance and metabolism for myocardial infarction therapy. Nat Commun. (2023) 14(1):6148. 10.1038/s41467-023-41700-0 37783697 PMC10545739

[25] Zhang Q Wang L Wang S Cheng H Xu L Pei G Signaling pathways and targeted therapy for myocardial infarction. Signal Transduct Target Ther. (2022) 7(1):78. 10.1038/s41392-022-00925-z 35273164 PMC8913803

[26] Chen L Li S Zhu J You A Huang X Yi X Mangiferin prevents myocardial infarction-induced apoptosis and heart failure in mice by activating the Sirt1/Foxo3a pathway. J Cell Mol Med. (2021) 25(6):2944–55. 10.1111/jcmm.16329 33523605 PMC7957271

[27] Whelan RS Konstantinidis K Wei AC Chen Y Reyna DE Jha S Bax regulates primary necrosis through mitochondrial dynamics. Proc Natl Acad Sci USA. (2012) 109(17):6566–71. 10.1073/pnas.1201608109 22493254 PMC3340068

[28] Cinar I Yayla M Tavaci T Toktay E Ugan RA Bayram P *In vivo* and *in vitro* cardioprotective effect of gossypin against isoproterenol-induced myocardial infarction injury. Cardiovasc Toxicol. (2022) 22(1):52–62. 10.1007/s12012-021-09698-3 34599475

[29] Lu G Jiang S Ashraf M Haider KH. Subcellular preconditioning of stem cells: mito-cx43 gene targeting is cytoprotective via shift of mitochondrial Bak and Bcl-Xl balance. Regen Med. (2012) 7(3):323–34. 10.2217/rme.12.13 22594326 PMC3380626

[30] Liang YJ Yang WX. Kinesins in Mapk Cascade: how kinesin motors are involved in the MAPK pathway? Gene. (2019) 684:1–9. 10.1016/j.gene.2018.10.042 30342167

[31] Frangogiannis NG. Regulation of the inflammatory response in cardiac repair. Circ Res. (2012) 110(1):159–73. 10.1161/circresaha.111.243162 22223212 PMC3690135

[32] Kologrivova I Shtatolkina M Suslova T Ryabov V. Cells of the immune system in cardiac remodeling: main players in resolution of inflammation and repair after myocardial infarction. Front Immunol. (2021) 12:664457. 10.3389/fimmu.2021.664457 33868315 PMC8050340

[33] Clark JE Sarafraz N Marber MS. Potential of P38-MAPK inhibitors in the treatment of ischaemic heart disease. Pharmacol Ther. (2007) 116(2):192–206. 10.1016/j.pharmthera.2007.06.013 17765316

[34] Lin CC Yang CC Wang CY Tseng HC Pan CS Hsiao LD Nadph oxidase/Ros-dependent Vcam-1 induction on Tnf-*Α*-challenged human cardiac fibroblasts enhances monocyte adhesion. Front Pharmacol. (2015) 6:310. 10.3389/fphar.2015.00310 26858641 PMC4729888

[35] Dewald O Zymek P Winkelmann K Koerting A Ren G Abou-Khamis T Ccl2/monocyte chemoattractant protein-1 regulates inflammatory responses critical to healing myocardial infarcts. Circ Res. (2005) 96(8):881–9. 10.1161/01.RES.0000163017.13772.3a 15774854

[36] Huang C Zhang Y Qi H Xu X Yang L Wang J. Myc is involved in genistein protecting against lps-induced myocarditis *in vitro* through mediating MAPK/JNK signaling pathway. Biosci Rep. (2020) 40(6):BSR20194472. 10.1042/bsr20194472 32515469 PMC7303346

[37] Wang G Ma L Wang B Gao F Li J Cai H Tanshinone iia accomplished protection against radiation-induced cardiomyocyte injury by regulating the P38/P53 pathway. Mediators Inflamm. (2022) 2022:1478181. 10.1155/2022/1478181 36046762 PMC9424041

[38] Shati AA. Doxorubicin-induces Nfat/Fas/Fasl cardiac apoptosis in rats through activation of calcineurin and P38 MAPK and inhibition of mtor signalling pathways. Clin Exp Pharmacol Physiol. (2020) 47(4):660–76. 10.1111/1440-1681.13225 31811646

[39] Raut GK Manchineela S Chakrabarti M Bhukya CK Naini R Venkateshwari A Imine stilbene analog ameliorate isoproterenol-induced cardiac hypertrophy and hydrogen peroxide-induced apoptosis. Free Radic Biol Med. (2020) 153:80–8. 10.1016/j.freeradbiomed.2020.04.014 32311492

[40] Hai-Yan Z Yong-Hong G Zhi-Yao W Bing X Ai-Ming W Yan-Wei X Astragalus polysaccharide suppresses the expression of adhesion molecules through the regulation of the P38 MAPK signaling pathway in human cardiac microvascular endothelial cells after ischemia-reperfusion injury. Evid Based Complement Alternat Med. (2013) 2013:280493. 10.1155/2013/280493 24302961 PMC3835432

[41] Shen E Chen RZ Yang YZ. Association of attenuating left ventricular remodeling in murine dilated cardiomyopathy with phosphorylation of p38 MAPK after interference by astragaloside. Chin J Pathophysiol. (2008) 24(1):64–7. 10.3321/j.issn:1000-4718.2008.01.016

[42] Du CS Yang RF Song SW Wang YP Kang JH Zhang R Magnesium Lithospermate B protects cardiomyocytes from ischemic injury via inhibition of Tab1-P38 apoptosis signaling. Front Pharmacol. (2010) 1:111. 10.3389/fphar.2010.00111 21607062 PMC3095368

[43] Ma H Zhang Y Wang J Guo W Hu G Xie S Palmatine attenuates lps-induced inflammatory response in mouse mammary epithelial cells through inhibiting Erk1/2, P38 and Akt/Nf-Кb signalling pathways. J Anim Physiol Anim Nutr. (2021) 105(1):183–90. 10.1111/jpn.13440 32865324

[44] Zhang Y Yin L Dong J Xia X. Soy isoflavones protect neuronal Pc12 cells against hypoxic damage through Nrf2 activation and suppression of P38 MAPK and Akt-Mtor pathways. Antioxidants. (2022) 11(10):2037. 10.3390/antiox11102037 36290760 PMC9598610

[45] Li HJ Wu NL Lee GA Hung CF. The therapeutic potential and molecular mechanism of isoflavone extract against psoriasis. Sci Rep. (2018) 8(1):6335. 10.1038/s41598-018-24726-z 29679037 PMC5910427

[46] Wu YJ Li SM Chen CL Chen ZR Chen JJ. Anti-inflammatory activity of pogostemon cablin: bioactive components and their modulation of MAPK and Nf-κb signaling pathway. Bioorg Chem. (2025) 161:108516. 10.1016/j.bioorg.2025.108516 40345124

[47] Guo S Zhang Q Li X Yu X Lan T Zhang W A network pharmacology-based approach to explore the molecular mechanism of Aidi injection against prostate cancer. Heliyon. (2024) 10(8):e29720. 10.1016/j.heliyon.2024.e29720 38681592 PMC11046112

